# Whole-Genome Resequencing Identifies the Molecular Genetic Cause for the Absence of a Gy5 Glycinin Protein in Soybean PI 603408

**DOI:** 10.1534/g3.117.039347

**Published:** 2017-06-06

**Authors:** Jason D. Gillman, Won-Seok Kim, Bo Song, Nathan W. Oehrle, Nilesh R. Tawari, Shanshan Liu, Hari B. Krishnan

**Affiliations:** * United States Department of Agriculture - Agricultural Research Service Plant Genetics Research Unit, Columbia, Missouri; † Plant Science Division, University of Missouri, Columbia, Missouri 65211,; ‡ Key Laboratory of Soybean Biology at the Chinese Ministry of Education, Northeast Agricultural University, Harbin 150030, China; § Computational and Systems Biology Group, Genome Institute of Singapore, Agency for Science, Technology and Research, 138672 Singapore

**Keywords:** soybean, *Glycine max*, glycinin, genomic resequencing, seed proteomics, single nucleotide variants (SNV), amino acid changes (AAC)

## Abstract

During ongoing proteomic analysis of the soybean (*Glycine max* (L.) Merr) germplasm collection, PI 603408 was identified as a landrace whose seeds lack accumulation of one of the major seed storage glycinin protein subunits. Whole genomic resequencing was used to identify a two-base deletion affecting *glycinin 5*. The newly discovered deletion was confirmed to be causative through immunological, genetic, and proteomic analysis, and no significant differences in total seed protein content were found to be due to the *glycinin 5* loss-of-function mutation *per se*. In addition to focused studies on this one specific *glycinin* subunit-encoding gene, a total of 1,858,185 nucleotide variants were identified, of which 39,344 were predicted to affect protein coding regions. In order to semiautomate analysis of a large number of soybean gene variants, a new SIFT 4G (Sorting Intolerant From Tolerated 4 Genomes) database was designed to predict the impact of nonsynonymous single nucleotide soybean gene variants, potentially enabling more rapid analysis of soybean resequencing data in the future.

Soybean (*Glycine max* L. Merr) is one of the most productive temperate crops, the seeds of which are a valuable source of protein and oil. Soybean seed proteins are largely (∼70%) comprised of two ancestrally related multi-gene seed storage protein families: glycinins and β-conglycinins (Nielsen *et al.* 1989; Krishnan 2001). Glycinins are encoded by five primary seed-expressed gene family members (and two pseudogenes) (Nielsen *et al.* 1989; Beilinson *et al.* 2002). Glycinin is synthesized as a larger precursor protein, which is post-translationally cleaved into acidic and basic subunits that are held together by a disulfide bond (Staswick *et al.* 1984). Glycinins are classified into groups I and II on the basis of their size and sequence homology (Nielsen *et al.* 1989). Group I comprises three subunits, A_1a_B_2_, A_1b_B_1b_, and A_2_B_1a_, whereas group II includes two subunits, A_3_B_4_ and A_5_A_4_B_3_ (Nielsen *et al.* 1989). The β-conglycinin gene family, though quite large, contains only three proteins (α’, α, and β protein subunits), which accumulate at appreciable levels in developing seeds (Thanh and Shibasaki 1978; Coates *et al.* 1985). The accumulation of the β-subunit of β-conglycinin is regulated by the availability of nitrogen and sulfur. Soybean plants grown in the presence of excess nitrogen have seeds that tend to have increased accumulation of the β-subunit of β-conglycinin (Gayler and Sykes 1985; Paek *et al.* 1997). Glycinin and β-conglycinin, on account of their abundance, are mainly responsible for the protein nutritive value of soybean seed. However, neither of these groups of proteins are rich in essential sulfur-containing amino acids (methionine and cysteine), though glycinins contain a relatively greater amount than β-conglycinins (Thanh and Shibasaki 1978).

Radiation-induced and spontaneous mutations that affect the accumulation of soybean seed proteins have been identified (Cho *et al.* 1989; Kitamura and Kaizuma 1981; Ogawa *et al.* 1995; Scallon *et al.* 1985; Hayashi *et al.* 1998; Kim *et al.* 2008, 2013; Takahashi *et al.* 1994). A soybean line that is devoid of both glycinins and β-conglycinins has also been developed by integrating multiple, distinct mutations (Takahashi *et al.* 2003). Interestingly, such a mutant was able to grow and reproduce normally, indicating that glycinins and β-conglycinins are dispensable and function only as storage proteins (Takahashi *et al.* 2003).

Cultivated soybean has almost no outcrossing (< 0.5%) in the field (Yoshimura *et al.* 2006). Owing to this fact, and the extreme genetic bottleneck events that occurred during domestication and cultivar development (Gizlice *et al.* 1994, 1996), soybean breeding can introduce artificial impacts on genetic diversity as compared to *G. max* landraces and/or wild *G. soja* lines. For example, the ancestors of all high-yielding North American soybean commercial lines examined have intact, expressed glycinin and β-conglycinin genes (Kim *et al.* 2008), yet mutants for several seed storage proteins exist in *G. max* landraces (such as PI 60348). In contrast, direct targets of domestication can feature either elevated or repressed diversity in landraces as compared to wild progenitors; *e.g.*, all known wild soybeans (*G. soja*) have black seedcoats, yet a range of seedcoat coloration is known to exist in *G. max* landraces (*e.g.*, green, yellow, brown, black, and red-brown, etc.) (Gillman *et al.* 2011), and almost all high-yielding soybean cultivars have been selected to have yellow seedcoats, with the only coloration present restricted to the hila (Valliyodan *et al.* 2016).

During the course of routine screening of seed from the USDA-GRIN soybean germplasm collection with immunological and proteomic analyses, a soybean line (PI 603408) was identified that produces seeds that lack a major seed storage protein, which was preliminary identified as a glycinin subunit based on protein size via SDS-PAGE analysis. The effect of this novel *gy5* loss-of-function allele was examined through a combination of proteomic and immunological analysis, NIRS spectroscopy, and classical genetic analysis. Very in-depth (∼59-fold coverage) genomic resequencing revealed a two-base deletion introducing a frameshift mutation in exon three of one specific glycinin gene (*gy5*), which was completely correlated with the absence of the glycinin protein subunit. In addition to determining the cause of the seed protein alteration, the deep genomic resequencing provided the opportunity to catalog the genetic variation in this virtually unexamined landrace. A new SIFT4G database, which uses a phylogenetic approach to predict the effect of single nucleotide changes on protein function, was developed and validated. The new genetic polymorphism data and the new database will likely be of use in future gene–variant and gene–function studies in soybean.

## Materials and Methods

### Plant materials

Seed of line PI 603408 was obtained from the USDA-GRIN collection. PI 603408 was donated by the Chinese Academy of Agricultural Sciences to the USDA-GRIN collection in 1998, marked as originating from Liaoning province. Seed of line “Patriot” was obtained from David Sleper of the University of Missouri. PI 603408 was crossed during the summer of 2013 to Patriot and to line “10/81b,” which is a *gy1a/gy1a* and *gy4/gy4* F_4:5_ line (PI 605781 B × Patriot) produced as part of a previous study (Kim *et al.* 2013). F_1_ seed from the two crosses were advanced two generations by single seed descent at a winter nursery. In 2014, F_3_ seeds from crosses were planted at South Farm Experimental farm (Columbia, MO, Latitude 38.908189, Longitude −92.278693, Mexico silt loam soil) and in 2015 at Hinkson Field (Columbia, MO, Latitude 38.928015, Longitude −92.351425, Haymond silt loam soil). Seeds were planted in 15 ft rows with a 3 ft gap between plots, a spacing of ∼2 in between plants, and row spacing of 30 in between rows. In 2014, individual plants were tagged and leaf tissue from individual plants sampled, freeze-dried, and crude DNA was isolated as previously described (Xin *et al.* 2003) or high-quality DNA was isolated with a DNeasy plant mini kit (QIAGEN, Valencia, CA). When plants had matured in 2014, individual plants were single plant threshed and seeds were stored at 4° and 39% relative humidity. Seed was harvested from individual plants in 2014 and 20 seeds were planted as five ft single plots in 2015, with spacing as described above. At maturity, each plot in 2015 was bulk harvested and stored at conditions described above until analysis.

### Genomic DNA resequencing analysis

High-quality DNA was isolated from ∼50 mg of lyophilized seedling leaf tissue from five PI 603408 plants using a DNeasy mini plant kit according to manufacturer’s recommendations (QIAGEN). Library construction and sequencing were performed by Global Biologics. (Columbia, MO). Briefly, genomic DNA was fragmented to 100–300 bp and two replicate Illumina genomic DNA resequencing libraries (100–300 bp insert) were prepared and sequenced using two entire lanes on a HISequation 2000 (2 × 100 bp, paired end). A total of 679,467,503 reads were collected. FASTQ files were imported into the CLC genomics workbench (version 9, QIAGEN), and each lane was separately mapped (due to memory limitations) to the Williams 82 reference genome W82.a2.v1 (Schmutz *et al.* 2010) using the following settings: mismatch cost of 2, insertion cost = 3, deletion cost = 3, length fraction = 0.5, similarity fraction = 0.8, auto-detection of paired distances = yes, and nonspecific matches = ignored (due to the ancestrally polyploid nature of soybean). The two lanes of mappings were then merged within the CLC genomics workbench. Full details on read mapping are located in Table 1.

**Table 1:** Details on genomic resequencing and read mapping

Reference (W82.a2.va)	Number of chromosomes	20
	Number of scaffolds	1,170
	Total reference length (bp)	978,495,272
Mapped reads (PI 603408)	Total read count	585,030,981
	Mean mapped read length (bp)	99.9
	Mapped total read length (bp)	58,431,063,227
	Mean coverage	59.1-fold
	Mean coverage excluding zero coverage regions	62.9-fold
	Mean paired end distance (bps)	180.5
Unaligned reads (PI 603408)	Total read count	94,436,522
	Unaligned percentage of all reads	16.1
Zero coverage regions (PI 603408)	Mean length (bp)	277.34
	Count	210,157
	Total length (bps)	58,284,351
	Percentage of genome (%)	6.0

### Variant calling and effect prediction

Sequence variants relative to the Williams 82 a2.v1 assembly were called using the basic variant detection function of CLC genomics workbench, using the following settings: ploidy = 2, ignore positions with coverage above = 100,000, ignore broken pairs = yes, ignore nonspecific matches = reads, minimum coverage = 10, minimum count = 7, minimum frequency = 50%, base quality filter = no, read direction filter = no, relative read direction filter = yes, and significance =1%. Variants predicted to result in amino acid changes (AAC) were predicted in the CLC genomics workbench using standard genetic code, and synonymous substitutions were filtered out.

### Prediction of impact of nonsynonymous AAC in PI 603408

SIFT 4G (Vaser *et al.* 2016) was used to predict the effect of nonsynonymous AAC on protein function. A custom database of predictions for all possible nonsynonymous SNPs was built using SIFT 4G for *G. max*, using the W82.a2.v1 assembly. SIFT outputs whether an AAC is deleterious or tolerated, and assigns a score. As described previously (Ng and Henikoff 2001), an amino acid substitution is predicted deleterious if the SIFT score is ≤ 0.05, and tolerated if the score is > 0.05; SIFT scores range from 0 to 1. However, for certain genes, no meaningful prediction could be made. On various datasets, SIFT’s accuracy ranges from 70.82 to 84.86% (Vaser *et al.* 2016). Complete details on the coding region polymorphisms identified, read count, and SIFT 4G predictions are present in Supplemental Material, File S1, and gene annotation information for W82.a2.v1 is located in File S2 (downloaded from www.soybase.org). CLC genomics identifies four classes of variant in coding regions: Single Nucleotide Variant substitutions (SNV), Multiple Nucleotide Variant substitutions (MNV), deletions, insertions, and “Replacements” (a term describing variants that combine deletions/insertions). Variants identified in PI 603408 resequencing are also provided in Variant Call Format files for all variants identified in PI603408 and for nonsynonymous substitutions in File S3 and File S4, respectively.

### DNA isolation Gy1, Gy4, and Gy5 genotyping assays

Genomic DNA was used with *Gy1* and *Gy4* genotyping assays as previously described (Kim *et al.* 2013).

A genotyping assay was designed to detect the two-base deletion affecting *gy5* in PI 603408, which relies on the introduction of “GC-tails” to a primer specific to each allele. A genotyping reaction has equal concentrations (0.5 μM in PCR reaction) of three primers: (1) a common primer that amplifies both alleles (5′-ACCATGACTCTTCTGCTGCTG-3′); (2) a primer specific for wild-type *Gy5* (5′-GCGGGCCTTGCTGGGAACCCAGATA**t**-3′); and (3) one specific for the gy5 deletion (5′-GCGGGCAGGGCGGCCTTGCTGGGAACCCAGATA**g**-3′). **Bold** indicates the allelic difference and underline indicates the GC-tail. In addition to primers, each reaction contained 10 µl of 2× QuantiTect SYBR Green (QIAGEN), 5–50 ng of genomic DNA, and water sufficient to bring the volume to 20 µl. Samples were amplified and analyzed on a Lightcycler 480 II instrument under the following conditions: 95° for 5 min followed by 35 cycles of 95° for 20 sec, 60° for 20 sec, and 72° for 20 sec, followed by a melting curve from 70° to 95°, with 20 readings taken every 1°.

### NIRS protein and oil determination

Seed moisture, oil, and protein were determined using ∼50 intact seed with a NIRS monochromator model FOSS 6500 (FOSS North America, Eden Prairie, MN) using the transport quarter cup (dimension 97 mm × 55 mm) and a calibration previously developed (La *et al.* 2014) by Andrew Scaboo of the University of Missouri. Seed oil and protein values were adjusted to 13% moisture content before statistical analysis.

### Statistical analysis

JMP Version 11 software (SAS Institute, Cary, NC) was used for one-way ANOVA tests. For any ANOVA tests that displayed significant differences at the *P* < 0.05 level, means were then compared using *t*-test *ad hoc* tests (α = 0.05 significance level cutoff). Full details on protein and oil data for the lines are present in File S5. Correlation between Gy5 protein band presence/absence and Gy5/gy5 genotypes are present in File S6.

### 1D gel electrophoresis

All chemicals described under electrophoresis and immunoblot analysis were obtained from Sigma Aldrich (St. Louis, MO). Soybean seeds were ground into a fine powder with a mortar and pestle and extracted with 1 ml of SDS-PAGE sample buffer containing protease inhibitor cocktail (Plant ProteaseArrest, G-Biosciences). The solution was centrifuged for 10 min to remove insoluble material at 16,100 × *g*. Supernatant was removed to a new tube and protein/buffer mixture was boiled for 5 min. A 10 µl aliquot of the boiled solution was used for electrophoresis. 1D separation was performed following the protocol of Laemmli (Laemmli 1970) using 13–15% T gels in a Mini250 apparatus (GE Healthcare). A constant current of 20 mA/gel was run with a typical run time of 1.2 hr. Following separation, gels were removed from the cassette, placed immediately in Coomassie R-250 staining solution, and destained with a 10% acetic acid solution.

### Immunoblot analysis

Seed proteins were resolved by SDS-PAGE as previously described, then electrophoretically transferred onto a 0.45 µm nitrocellulose membrane. Membranes were then incubated with 5% nonfat dry milk/TBS buffer (10 mM Tris-HCl, pH 7.5 and 500 mM NaCl) for 1 hr at room temperature. Following this step, membranes were incubated overnight with antibodies that had been diluted 1:20,000 in TBST (TBS with 3% nonfat dry milk containing 0.2% Tween 20). The following day, membranes were washed 3× with TBST and incubated with goat anti-rabbit IgG-horseradish peroxidase conjugate that had been diluted 1:20,000 in TBST. Proteins that reacted with antibodies were detected using a SuperSignal West Pico chemiluminescence kit (Pierce).

### 2D electrophoresis

Soybean seeds were ground to a fine powder and a 250 mg subsample was used for extraction with a cold mortar/pestle and 5 ml of extraction buffer [0.9 M sucrose, 0.1 M Tris-Cl (pH 8.8), and 0.4% 2-mercaptoethanol] as well as 50 µl of Plant ProteaseArrest (G-Biosciences). Samples were ground for 5 min until a liquid consistency was reached and removed to a 15 ml tube. Next, 5 ml of Tris-equilibrated phenol was added and phase separation was achieved using centrifugation (5000 × *g*, 20 min) via a swing-bucket rotor. The upper phenolic phase was removed to a fresh tube and proteins were precipitated using 10 volumes of freshly prepared 100% methanol with 0.1 M ammonium acetate for 2 hr at −80°, followed by centrifugation (12000 × *g*, 20 min) at 4°. The resulting protein pellet was thoroughly resuspended in freshly prepared ice-cold solution (100% methanol, 0.1 M ammonium acetate, and 10 mM DTT). Washing was repeated 3× with the same solution and 3× with freshly prepared 100% acetone containing 10 mM DTT (ice cold). Incubations of 30 min at −20° were followed by centrifugation at 12000 × *g* for 10 min at 4° between each wash.

400 µg of protein sample was loaded per IEF strip using in-gel rehydration. Linear gradient, 13 cm IPG strips (GE Healthcare) were brought to a rehydration volume of 250 µl with 7 M urea, 2 M thiourea, 1% CHAPS, 2% C7BzO, 5% glycerol, and 2.2% 2-HED, containing protein sample. IEF strips were equilibrated (post-IEF) with 5% SDS in a urea-based solution (0.05 M Tris-Cl pH 8.8, 6 M urea, 30% glycerol, and 0.1% bromophenol blue) containing 2% DTT for 10 min, and again but with 2.5% iodoacetamide for 10 min. Focused strips were placed onto a medium format 15% T vertical second dimension and secured into place with a warm 1% agarose SDS-PAGE running buffer solution (0.2% SDS). Gels were run at an initial 10 mA/gel for 1 hr and followed by 25 mA/gel for 3 hr. Gels were immediately removed and fixed for 30 min in 5:4:1 methanol:water:acetic acid solution, followed by two brief rinses in water. Finally, gels were stained in a Coomassie G-250 solution overnight.

### 2D image acquisition and analysis

1DE and 2DE Coomassie-stained gels were destained with multiple changes of ultrapure H_2_O to remove nonspecific background. Gels were scanned separately using an Epson V700 Perfection scanner under control of Adobe Photoshop. 1DE Images were analyzed using Phoretix-Quant (TotalLab, Newcastle upon Tyne, UK) for band identification, location, and R_f_, and 2DE images were analyzed using Delta2D v3.6 (Decodon, Greifswald, Germany); spot location calibration, and normalized % spot volume data were obtained using a technique known as differential gel imaging and analysis.

### Data availability

The authors state that all data necessary for confirming the conclusions presented in the article are either fully represented within this article, archived in a sequence read archive, or are present within manuscript supplemental files. FASTQ files and BAM files have been archived at the NCBI sequence read archive under project PRJNA343126 and accession SRP090021.

## Results and Discussion

### SDS-PAGE analysis of seed From PI 603408 confirmed the 42 kDa protein to be a Gy5 subunit

**Figure 1:**
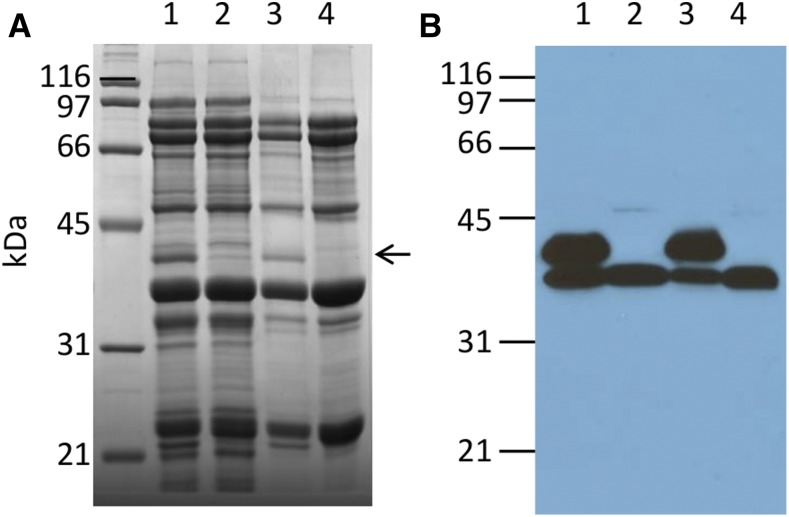
SDS-PAGE analysis of mature seed from lines Patriot and PI 603408. (A) SDS-PAGE analysis of total seed proteins (samples 1 and 2) or calcium carbonate enrichment of seed storage proteins (samples 3 and 4), and (B) immunoblot analysis of total seed proteins (samples 1 and 2) or calcium carbonate enrichment of seed storage proteins (samples 3 and 4) with anti-glycinin 4/5 antibodies. Samples 1 and 3 are from seed of cultivar Patriot and samples 2 and 4 are from seed of PI 603408. Arrow indicates protein band polymorphism between genotypes. SDS-PAGE, sodium dodecyl sulfate polyacrylamide gel electrophoresis.

As part of ongoing investigations to find soybean lines with altered seed protein composition and/or content, PI 603408 was identified as a line that lacked a single protein band (Figure 1, arrow), as determined by 1D SDS-PAGE analysis. The molecular weight of this protein band was estimated to be 42 kDa. It has previously been demonstrated that soybean seed proteins can be preferentially precipitated from total seed extracts by the addition of calcium (Krishnan *et al.* 2009). Based on previous proteomic studies, the protein absent in PI 603408 was tentatively identified as Glycinin 5 (Glyma13g18450.1/Glyma.13g123500.1). Further confirmation was obtained by immunoblot analysis using antibodies raised against Gy5 protein (Figure 1B). Western blot analysis revealed that anti-Gy5 antibodies reacted strongly against two proteins with molecular weights of 42 and 40 kDa. The identity of the 40 kDa protein most likely represents the closely related glycinin gene family member Gy4. In contrast, soybean PI 603408 failed to accumulate the 42 kDa protein (Figure 1B), indicating that the 42 kDa protein is the Gy5 subunit.

### Establishing a SIFT 4G database to assist evaluation of mutations identified by genome resequencing of PI 603408

In order to determine the molecular genetic cause for the protein band absence, an attempt was made to clone the *gy5* gene by simple PCR utilizing several primer combinations. Glycinin-specific PCR amplification products of the expected sizes were obtained, yet sequence analysis revealed that all amplified sequences corresponded only to the *Gy4* gene. This result is not surprising given the very high sequence homology of *Gy4* and *Gy5* genes (Nielsen *et al.* 1989). PI 603408 represents a completely untapped reservoir of genetic diversity, as well as a novel *gy5* mutation. For both these reasons, the genome of PI 603408 was resequenced using two distinct Illumina genomic DNA resequencing libraries, which were mapped to the Williams 82 a2.v1 assembly reference sequence (Schmutz *et al.* 2010) using a very high level of sequencing depth (∼59-fold coverage). This revealed a total of 1,858,185 nucleotide variants, which included: 1,542,261 SNV, 44,162 MNV, 129,082 insertions, 134,457 deletions, and 8223 replacements. A subset of the newly identified polymorphisms were predicted to result in AAC and/or impact protein coding genomic regions: 34,470 SNV, 1490 MNV, 1576 insertions, 1708 deletions (including the *gy5* two-base deletion), and 100 replacements (more complicated rearrangements or deletion/insertions). Collectively, an estimated 39,344 nonsynonymous alterations were found in 13,590 genes, including 2285 predicted frameshift mutations and 241 stop codon gains. As validation for the small read mapping results, two known mutations were also identified: (1) a photoperiod response gene E2 (Glyma.10G221500) in PI 603408 has a mutation [1582A > T, Lys528*(Watanabe *et al.* 2011)] which truncates the open reading frame by converting a lysine residue to a stop codon; and (2) a single-base nucleotide deletion affecting the Glyma.01G214600 (182delT, Leu61fs) *GmSGR2*/*D1* gene, which has been shown to be associated with retention of chlorophyll (*i.e.*, staygreen) in soybean (Fang *et al.* 2014; Nakano *et al.* 2014).

The effect of a frameshift event on protein function/accumulation, for example due to deletion or insertion, is relatively easy to predict. Unfortunately, these events are relatively rare in natural populations; SNV-based amino acid substitution events are far more common. Two major hurdles in whole-scale genomic resequencing studies are: (1) analysis of less extreme polymorphism-predicted effects in a phylogenetic context; and (2) the need for automation owing to the extremely large number of variants identified in such studies. Toward this end, a custom SIFT 4G database was created for the current version of the soybean genome (W82.a2.v1), which can evaluate and predict the effect of SNV. This database will be publically available for download and open use by anyone (http://sift.bii.a-star.edu.sg/sift4g/). Out of 22,877 SNVs predicted by SIFT 4G, 3316 (of which 975 have low-confidence predictions) were predicted to be damaging, whereas 16,837 were predicted to be tolerated. The effect of another 2724 nonsynonymous substitutions could not be predicted by SIFT4G. Full details on variants identified, sequencing read counts, and the predicted effects of nonsynonymous substitutions is available in File S1.

### Identification of loss-of-function mutations in seed-expressed genes

The major seed-expressed glycinin and conglycinin genes in the genomic resequencing mapping were examined for allelic differences in PI 603408 (Table 2) and a number of putative variants were identified. A two-base deletion was found within exon 3 of Glyma13g18450/Glyma.13g123500 *Glycinin 5* (584_585delTA), which introduces a frameshift mutation (Ile195fs, within exon 3). We also identified a number of nonsynonymous variants in other glycinin-encoding genes, though none were predicted to affect protein accumulation, as determined by SIFT analysis. (Table 2, full details of all nonsynonymous variants are in File S1). *A priori*, it might have been anticipated that mapping of small 100–200 bp sequencing reads to the complicated polyploid soybean genome could result in difficulties, particularly with the large multi-gene glycinin and conglycinin families. However, this does not appear to have been a significant hindrance.

**Table 2:** Polymorphisms detected in *glycinin* and β*-conglycinin* genes in PI 603408 as compared to the Williams 82 a2.v1 genome

Family	Wm82.a2.v1	Wm81.a1.v1.1	Gene Name	Seed Protein(s)	Variants Identified	Amino Acid Change	SIFT Prediction	SIFT Score	Seed Expression
β-conglycinin	Glyma.10g028300	Glyma10g03390	*CG-1*	CG-α′-subunit	149C > A	Pro50Gln	Tolerated	1	Seed expressed
	Glyma.10g028300	Glyma10g03390	*CG-1*	CG-α′-subunit	192A > T	Arg64Ser	Tolerated	0.654	Seed expressed
	Glyma.10g246300	Glyma10g39150	*CG-1*	CG-α′-subunit	1280G > A	Arg427His	Tolerated	0.223	Seed expressed
	No correspondence	Glyma10g39161	*—*	NA	No correspondence	—	—	—	—
	Glyma.10g246500	Glyma10g39170	*NA*	NA	—	—	—	—	—
	Glyma.20g146200	Glyma20g28460	*CG-4*	CG-β-subunit	108G > T	Leu36Phe	Tolerated	1	Seed expressed
	Glyma.20g148200	Glyma20g28640	*CG-4*	CG-β-subunit	—	—	—	—	
	Glyma.20g148400	Glyma20g28650	*CG-2*	CG-α-subunit	—	—	—	—	Seed expressed
	Glyma.20g148300	Glyma20g28660	*CG-3*	CG-α-subunit	—	—	—	—	Seed expressed
Glycinin	No correspondence	Glyma03g32010	*Gy6a**^a^*	NA	No correspondence	—	—	—	Pseudogene
	No correspondence	Glyma03g32020	*Gy2*	A_2_ B_2_	No correspondence	—	—	—	Seed expressed
	Glyma.03g163500	Glyma03g32030	*Gy1*	A_1a_ B_1a_	604_606delCATinsTCC	His202Ser	Tolerated	0.74	Seed expressed
	Glyma.03g163500	Glyma03g32030	*Gy1*	A_1a_ B_1a_	151C > T	Leu51Phe	Tolerated	0.229	Seed expressed
	Glyma.10g037100	Glyma10g04280	*Gy4*	A_4_ A_5_ B_3_	—	—	—	—	Seed expressed
	Glyma.13g123500	Glyma13g18450	*Gy5*	A_3_ B_4_	584_585delTA	Ile195fs	NA*^b^*	NA*^b^*	Seed expressed
	Glyma.19g164800	Glyma19g34770	*Gy7**^a^*	NA	1031T > A	Val344Glu	Tolerated	0.951	Pseudogene
	Glyma.19g164800	Glyma19g34770	*Gy7**^a^*	NA	910G > A	Asp304Asn	Tolerated	0.085	Pseudogene
	Glyma.19g164900	Glyma19g34780	*Gy3*	A_1b_ B_1b_	—	—	—	—	Seed expressed

SIFT, Sorting Intolerant From Tolerated; NA, not applicable; 4G, four genomes.

a Pseudogene.

b SIFT 4G does not interpret multiple nucleotide polymorphisms.

### Molecular marker assays confirmed gy5 mutation is causative for absence of Gy5 subunit in seed of PI 603408

**Figure 2:**
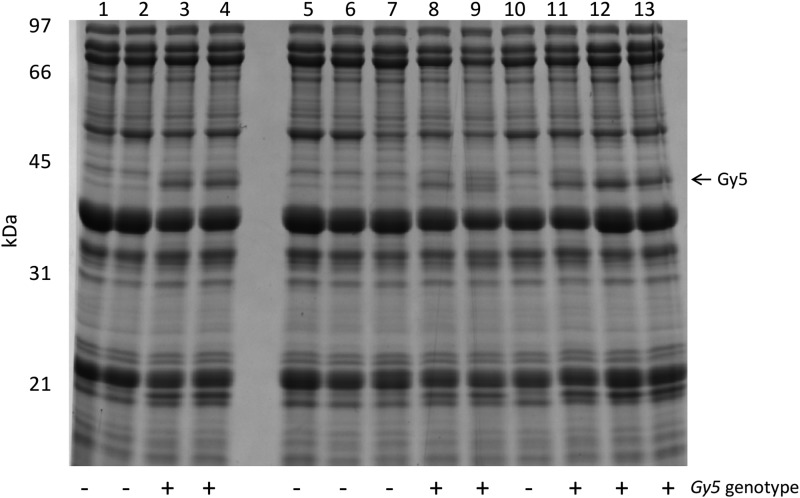
SDS-PAGE analysis of progeny of a F_3:4_ Patriot × PI 603408 cross. (A) SDS-PAGE analysis of total seed proteins. Samples 1 and 2 are seed from PI 603408, samples 3 and 4 are seed from line Patriot. Samples 5–13 are selected progeny from a Patriot × PI 603408 cross. The genotype of the plant that produced seed is indicated below the gel image: “+” indicates homozygosity for wild-type alleles and “−” indicates homozygosity for mutant alleles. SDS-PAGE, sodium dodecyl sulfate polyacrylamide gel electrophoresis.

**Figure 3:**
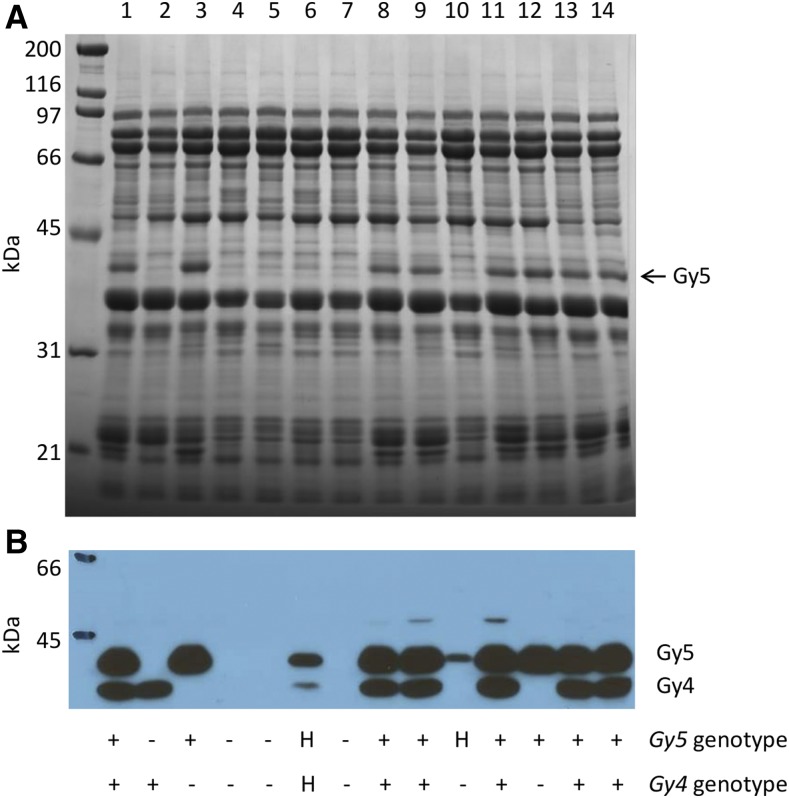
SDS-PAGE and immunoblot analysis of F_4:5_ progeny of a 10/81b × PI 603408 cross. (A) SDS-PAGE analysis of total seed proteins and (B) immunoblot analysis of total seed proteins with anti-glycinin 5 antibodies. Samples 1, 13, and 14 are from line Patriot and sample 2 is seed from PI 603408 plants. Samples 3–12 are selected progeny from a 10/81b × PI 603408 cross. The genotype of plant that produced seed is indicated below figure: “+” indicates homozygosity for wild-type alleles, “H” indicates heterozygosity, and “−” indicates homozygosity for mutant alleles. SDS-PAGE, sodium dodecyl sulfate polyacrylamide gel electrophoresis.

The presence of multiple glycinin mutations could have the potential to result in pleiotropic effects on seed composition, particularly seed protein and/or oil content. In order to track the novel two-nucleotide *gy5* deletion, a GC-tail molecular marker (Wang *et al.* 2005) was developed and tested on a segregating population derived from a cross between PI 603408 and a public cultivar, Patriot. DNA from individual F_3_ plants were genotyped, F_3:4_ seed from single plant threshes were harvested, and a subset analyzed via SDS-PAGE (Figure 2 and Figure 3) and with an NIRS calibration able to predict soybean seed protein and seed oil. Seed constituents from F_4:5_ lines planted in a completely randomized field experiment in 2015 were also analyzed by SDS-PAGE analysis and NIRS.

The *gy5* molecular marker assay was 95% accurate (69/74) in predicting the presence of the glycinin subunit using a crude DNA preparation (Xin *et al.* 2003); a small number of heterozygotes were mistakenly called as homozygous *gy5/gy5*. When DNA was reisolated using a DNeasy plant mini kit (QIAGEN), the *gy5* assay was 100% accurate (73/73). No evidence for segregation distortion was observed in either cross (data not shown), and no significant differences were noted between any genotypic classes (*gy5/gy5*; *gy5/WT*; *WT/WT*) for protein in 2014 or 2015 (Table 3). A very slight (< 0.1%) reduction in seed oil was noted for the homozygote mutant line (*gy5/gy5*) relative to the homozygote (*WT/WT*) genotypic class for the cross of (Patriot × PI 605781 B); this may indicate slight linkage drag.

**Table 3:** Near-Infrared Reflectance Spectroscopy seed compositional analysis of effects of the glycinin 5 mutation in two populations: Patriot × PI603408 (in 2014/2015) and 10/81b × PI603408 (in 2015)

				# Individuals in Each Genotypic Class			Mean (% Seed) + SE*^a^*^,^*^b^*			ANOVA Yr		ANOVA *Gy5* Genotype	
Cross	Yr	Gen.	Seed Trait	*gy5/gy5*	*gy5/WT*	*WT/WT*	*gy5/gy5*	*gy5/WT*	*WT/WT*	*F*-Value	P	*F*-Value	P
Patriot	2014	F4	Seed oil	21	21	21	17.2 + 0.1 A	17.6 + 0.1 A	17.7 + 0.1 A	0.347	0.557 NS	3.768	0.025*
×	2015	F5		38	28	34							
PI603408	2014	F4	Protein	21	21	21	35 + 0.3 A	35.2 + 0.4 A	34.8 + 0.3 A	1.425	0.234 NS	0.3363	0.715 NS
	2015	F5		38	28	34							
10/81b × PI603408	2015	F5	Protein	18	—	13	34.3 + 0.6 A	—	34.2 + 0.8 A	—		0.009	0.925 NS
			Seed oil				17.4 + 0.3 A	—	17.4 + 0.3 A	—		0.009	0.925 NS

* indicates ANOVA result *P* < 0.05. Yr, year; Gen., generation; WT, wild-type; NS, insignificant differences between means; NIRS, .

a NIRS results were adjusted to 13% moisture.

b Samples with identical letters are significantly different by *t*-test, (α = 0.05).

### 2D comparative analysis of the gy1/gy4/gy5 homozygote line in comparison to Patriot revealed substantial changes in only Gy1/Gy4/Gy5 proteins

**Figure 4:**
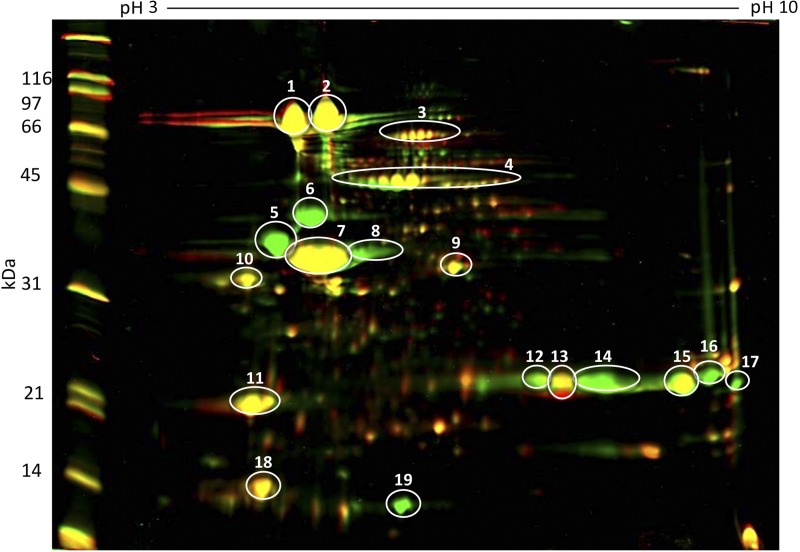
Overlay of two separate 2D gels of soybean seed proteins using Delta2D software. Isoelectric focusing (pI 3–10) followed by second dimension SDS-PAGE resulted in the separation of seed proteins and visualization of those proteins using Coomassie Blue. Gels were scanned and the resulting images were assigned two different colors (green = Patriot and red = gy1/gy4/gy5 mutant) in order to visualize the differences between the two. Delta2D software provides an overlay of both, with spot matching, where yellow demonstrates similar protein quantities in each. Green color demonstrates absence of that particular protein species in the gy1/gy4/gy5 mutant. Spots 1, 2, and 4 represent the 7S β-conglycinin subunits and spots 5–10, 11–17, and 19 represents the different glycinin subunits. Spots 3, 11, and 18 are the sucrose-binding proteins, KTi and BBi, respectively. SDS-PAGE, sodium dodecyl sulfate polyacrylamide gel electrophoresis.

In previous work, we had begun to integrate two distinct glycinin mutations (*gy1/gy1* and *gy4/gy4*) in the background of soybean cultivar Patriot (Kim *et al.* 2013). To develop a soybean line lacking Gy1/Gy4/Gy5 proteins, a second cross was made with PI 603408 crossed to a line derived from Patriot × PI 605781 B homozygous for two glycinin mutations (*gy1/gy1* and *gy4/gy4*, ∼50% genome from PI 605781 B and ∼50% genome from Patriot). Due to the presence of three segregating genes and a smaller-sized population, the second set of RILs was only evaluated using F_4:5_ seed produced in 2015. No significant differences in seed oil or seed protein were noted between these lines in 2015 (Table 3); however, the loss of three different glycinin subunits has the potential to have proteome rebalancing pleiotropic effects on seed proteome composition (Schmidt and Herman 2008; Schmidt *et al.* 2011; Herman 2014). To evaluate this possibility, seed proteins were separated in two-dimensions (isoelectric focusing and SDS-PAGE) and compared with seed of a *gy1/gy4/gy5* homozygous mutant in relation to Williams 82 seed (Figure 4). The absence of three glycinin protein subunits (as well as their precursors) was confirmed. Aside from the absence of Gy1/Gy4/Gy5 protein spots and slight differential protein band migration (presumably due to different isoforms), the seed proteomes were very comparable; little or no significant changes in β-conglycinin, Bowman–Birk proteinase inhibitors, Kunitz Trypsin Inhibitors, or Gy2 and Gy3 protein levels were noted (Figure 4).

Proteome rebalancing has been demonstrated to dramatically alter protein levels in lines with multiple genes whose expression has been reduced through RNAi (Schmidt *et al.* 2011). We saw no evidence of this phenomenon in the triple mutant genetic material. However, the lines in this study are not isogenic as is the case in RNAi studies; a substantial backcrossing effort would be required to generate true near-isogenic lines. Relatively large variances were noted for seed protein and oil *gy1/gy4/gy5* genotypic RIL categories in comparison to parental line seeds (File S5), which we attribute to multiple independent genetic loci controlling seed traits between our parental lines. Although there appears to be no significant effect of the *gy5* mutation alone (Table 3), there may be a small decrease in total protein in the triple mutant materials that is hidden by other genetic factors (or alternatively, small increases in other seed storage proteins). As a result, we cannot conclusively confirm or refute the proteome rebalancing hypothesis (Schmidt *et al.* 2011) with our genetic material at present.

### Conclusions

Through proteomic analysis, an unimproved soybean landrace, PI 603408, was identified whose seeds lacked a glycinin protein subunit. Through whole genomic resequencing at a very high coverage depth (∼59-fold), the molecular genetic cause was determined to be a two-base deletion that introduces a frameshift mutation in *Glycinin 5*. This was confirmed by cosegregation of the mutation with the absence of Gy5 protein in two independent segregating populations. The two-nucleotide deletion was found to have no significant effect on seed protein in field experiments over 2 yr. In addition, a total of 1,858,185 nucleotide variants were detected by resequencing PI 603408, as compared to the reference genome Williams 82, and 39,344 variants were predicted to result in coding region changes, affecting 13,590 genes. A newly developed SIFT 4G database was used to predict the effect of the SNV using ancestral conservation scoring across a range of diverse species. We anticipate that the new SIFT 4G database, as well as the extremely high coverage depth (average 59.1-fold) resequencing information for PI 603408, will prove useful in future soybean gene diversity and gene function studies.

## Supplementary Material

Supplemental material is available online at www.g3journal.org/lookup/suppl/doi:10.1534/g3.117.039347/-/DC1.

## References

[bib1] Beilinson V. Chen Z. Shoemaker R. Fischer R. Goldberg R., 2002 Genomic organization of glycinin genes in soybean. Theor. Appl. Genet. 104(6): 1132–1140. 12582623 10.1007/s00122-002-0884-6

[bib2] Cho T. J. Davies C. S. Nielsen N. C., 1989 Inheritance and organization of glycinin genes in soybean. Plant Cell 1(3): 329–337. 12359892 10.1105/tpc.1.3.329 PMC159765

[bib3] Coates J. B. Medeiros J. S. Thanh V. H. Nielsen N. C., 1985 Characterization of the subunits of β-conglycinin. Arch. Biochem. Biophys. 243(1): 184–194. 3840670 10.1016/0003-9861(85)90787-8

[bib4] Fang C. Li C. Li W. Wang Z. Zhou Z., 2014 Concerted evolution of D1 and D2 to regulate chlorophyll degradation in soybean. Plant J. 77(5): 700–712. 24372721 10.1111/tpj.12419

[bib5] Gayler K. R. Sykes G. E., 1985 Effects of nutritional stress on the storage proteins of soybeans. Plant Physiol. 78(3): 582–585. 16664286 10.1104/pp.78.3.582 PMC1064779

[bib6] Gillman J. D. Tetlow A. Lee J. D. Shannon J. G. Bilyeu K., 2011 Loss-of-function mutations affecting a specific Glycine max R2R3 MYB transcription factor result in brown hilum and brown seed coats. BMC Plant Biol. 11(1): 115. 22070454 10.1186/1471-2229-11-155 PMC3229458

[bib7] Gizlice Z. Carter T. E. Jr. Burton J. W., 1994 Genetic base for North American public soybean cultivars released between 1947 and 1988. Crop Sci. 34(5): 1143–1151.

[bib8] Gizlice Z. Carter T. E. Jr. Gerig T. M. Burton J. W., 1996 Genetic diversity patterns in North American public soybean cultivars based on coefficient of parentage. Crop Sci. 36(3): 753–765.

[bib9] Hayashi M. Harada K. Fujiwara T. Kitamura K., 1998 Characterization of a 7S globulin-deficient mutant of soybean (Glycine max (L.) Merrill). Mol. Gen. Genet. 258(3): 208–214. 9645426 10.1007/s004380050724

[bib10] Herman E. M., 2014 Soybean seed proteome rebalancing. Front. Plant Sci. 5: 437. 25232359 10.3389/fpls.2014.00437 PMC4153022

[bib11] Kim W.-S. Ho H. J. Nelson R. L. Krishnan H. B., 2008 Identification of several gy4 nulls from the USDA soybean germplasm collection provides new genetic resources for the development of high-quality Tofu cultivars. J. Agric. Food Chem. 56(23): 11320–11326. 18991447 10.1021/jf801831w

[bib12] Kim W.-S. Gillman J. D. Krishnan H. B., 2013 Identification of a plant introduction soybean line with genetic lesions affecting two distinct glycinin subunits and evaluation of impacts on protein content and composition. Mol. Breed. 32(2): 291–298.

[bib13] Kitamura K. Kaizuma N., 1981 Mutant strains with low loft subunits of 7S globulin in soybean (Glycine max Merr.) seed. Japan. J. Breed. 31(4): 353–359.

[bib14] Krishnan H. B., 2001 Biochemistry and molecular biology of soybean seed storage proteins. J. New Seeds 2(3): 1–25.

[bib15] Krishnan H. B. Oehrle N. W. Natarajan S. S., 2009 A rapid and simple procedure for the depletion of abundant storage proteins from legume seeds to advance proteome analysis: a case study using Glycine max. Proteomics 9(11): 3174–3188. 19526550 10.1002/pmic.200800875

[bib16] La T. C. Pathan S. M. Vuong T. Lee J.-D. Scaboo A. M., 2014 Effect of high-oleic acid soybean on seed oil, protein concentration, and yield. Crop Sci. 54(5): 2054–2062.

[bib17] Laemmli U. K., 1970 Cleavage of structural proteins during the assembly of the head of bacteriophage T4. Nature 227(5259): 680–685. 5432063 10.1038/227680a0

[bib18] Nakano M. Yamada T. Masuda Y. Sato Y. Kobayashi H., 2014 A green-cotyledon/stay-green mutant exemplifies the ancient whole-genome duplications in soybean. Plant Cell Physiol. 55(10): 1763–1771. 25108243 10.1093/pcp/pcu107

[bib19] Ng P. C. Henikoff S., 2001 Predicting deleterious amino acid substitutions. Genome Res. 11(5): 863–874. 11337480 10.1101/gr.176601 PMC311071

[bib20] Nielsen N. C. Dickinson C. D. Cho T. J. Thanh V. H. Scallon B. J., 1989 Characterization of the glycinin gene family in soybean. Plant Cell 1(3): 313–328. 2485233 10.1105/tpc.1.3.313 PMC159764

[bib21] Ogawa T. Bando N. Tsuji H. Nishikawa K. Kitamura K., 1995 Alpha-subunit of beta-conglycinin, an allergenic protein recognized by IgE antibodies of soybean-sensitive patients with atopic dermatitis. Biosci. Biotechnol. Biochem. 59(5): 831–833. 7787297 10.1271/bbb.59.831

[bib22] Paek N. C. Imsande J. Shoemaker R. C. Shibles R., 1997 Nutritional control of soybean seed storage protein. Crop Sci. 37(2): 498–503.

[bib23] Scallon B. Thanh V. H. Floener L. A. Nielsen N. C., 1985 Identification and characterization of DNA clones encoding group-II glycinin subunits. Theor. Appl. Genet. 70: 510–519. 24253061 10.1007/BF00305984

[bib24] Schmidt M. A. Herman E. M., 2008 Proteome rebalancing in soybean seeds can be exploited to enhance foreign protein accumulation. Plant Biotechnol. J. 6(8): 832–842. 18694455 10.1111/j.1467-7652.2008.00364.x

[bib25] Schmidt M. A. Barbazuk W. B. Sandford M. May G. Song Z., 2011 Silencing of soybean seed storage proteins results in a rebalanced protein composition preserving seed protein content without major collateral changes in the Metabolome and Transcriptome. Plant Physiol. 156(1): 330–345. 21398260 10.1104/pp.111.173807 PMC3091051

[bib26] Schmutz J. Cannon S. B. Schlueter J. Ma J. Mitros T., 2010 Genome sequence of the palaeopolyploid soybean. Nature 463: 178–183. 20075913 10.1038/nature08670

[bib27] Staswick P. E. Hermodson M. A. Nielsen N. C., 1984 The amino acid sequence of the A2B1a subunit of glycinin. J. Biol. Chem. 259(21): 13424–13430. 6541652

[bib28] Takahashi K. Banba H. Kikuchi A. Ito M. Nakamura S., 1994 An induced mutant line lacking the alpha;-subunit of beta;-conglycinin in soybean (Glycine max (L.) Merrill). Japan. J. Breed. 44(1): 65–66.

[bib29] Takahashi M. Uematsu Y. Kashiwaba K. Yagasaki K. Hajika M., 2003 Accumulation of high levels of free amino acids in soybean seeds through integration of mutations conferring seed protein deficiency. Planta 217(4): 577–586. 12684787 10.1007/s00425-003-1026-3

[bib30] Thanh V. H. Shibasaki K., 1978 Major proteins of soybean seeds. Subunit structure of. beta.-conglycinin. J. Agric. Food Chem. 26(3): 692–695.

[bib31] Valliyodan B. Dan Q. Patil G. Zeng P. Huang J., 2016 Landscape of genomic diversity and trait discovery in soybean. Sci. Rep. 6: 23598. 27029319 10.1038/srep23598 PMC4814817

[bib32] Vaser R. Adusumalli S. Leng S. N. Sikic M. Ng P. C., 2016 SIFT missense predictions for genomes. Nat. Protoc. 11(1): 1–9. 26633127 10.1038/nprot.2015.123

[bib33] Wang J. Chuang K. Ahluwalia M. Patel S. Umblas N., 2005 High-throughput SNP genotyping by single-tube PCR with Tm-shift primers. Biotechniques 39(6): 885–893. 16382908 10.2144/000112028

[bib34] Watanabe S. Xia Z. Hideshima R. Tsubokura Y. Sato S., 2011 A map-based cloning strategy employing a residual heterozygous line reveals that the GIGANTEA gene is involved in soybean maturity and flowering. Genetics 188: 395–407. 21406680 10.1534/genetics.110.125062 PMC3122305

[bib35] Xin Z. Velten J. P. Oliver M. J. Burke J. J., 2003 High-throughput DNA extraction method suitable for PCR. Biotechniques 34: 820–826. 12703307 10.2144/03344rr04

[bib36] Yoshimura Y. Matsuo K. Yasuda K., 2006 Gene flow from GM glyphosate-tolerant to conventional soybeans under field conditions in Japan. Environ. Biosafety Res. 5(3): 169–173. 17445512 10.1051/ebr:2007003

